# Is Defensive Behavior a Subtype of Prosocial Behaviors?

**DOI:** 10.3389/fpsyg.2021.678370

**Published:** 2021-06-23

**Authors:** Alessandra Geraci, Laura Franchin

**Affiliations:** Department of Psychology and Cognitive Sciences, University of Trento, Rovereto, Italy

**Keywords:** prosociality, emotional distress, evaluation, sociomoral development, defensive behaviors

## Introduction

Defensive behavior is defined as a behavioral response to threatening situations for survival and body safety of oneself and others (Pulkkinen, 1987; Wrangham, 2018). Defending is a prosocial behavior defined by the intent to help victimized individuals (Eisenberg and Spinrad, 2014; Lambe and Craig, 2020). Lambe and Craig (2020) proposed that defensive behavior can be conceptualized as a multidimensional behavior, suggesting that solution-focused and aggressive defending are ways in which individuals defend directly while reporting to authority and comforting are ways in which people defend indirectly supporting the victim. Together, these four behaviors reflect different ways in which individuals can defend others from victimization. Reflecting on the social value of defensive behavior, some questions come to mind and need to be answered.

Generally, prosocial behaviors are defined as intervening actions that are preceded by the direct observation or inference of another's a negative state (Dunfield and Kuhlmeier, 2013; Warneken, 2013; Dunfield, 2014; Eisenberg and Spinrad, 2014; Tomasello, 2019). Prosocial behaviors rest on the ability (i) to recognize that another is having a negative experience, (ii) to understand what an appropriate response would entail, and (iii) to act with the motivation to intervene. Dunfield (2014) claimed that within the general domain of prosocial behaviors there are three distinct types (helping, sharing, and comforting), which are responses to three negative states: *instrumental need* when individuals cannot complete a goal-directed action; *material desire*, when individuals do not have access to resources; *emotional distress* when individuals experience a negative emotional state. Each of these negative states can be alleviated by a different prosocial behavior, such as helping, sharing, or comforting, respectively. These prosocial behaviors rely on different social-cognitive assessments, such as goals for the helping, desires for the sharing, and emotions for the comforting. These three types of behavior show unique ages of onset, dissociable developmental trajectories, and distinct associations with individual difference factors early in life. Recent research has emphasized the multidimensional nature of prosocial behavior and has fueled the current debate on the psychological mechanisms underlying prosocial actions in early childhood (Dunfield, 2014; Paulus, 2014, 2018). According to Dunfield's model (2014), we believe that defending action can be among prosocial behaviors as a subtype for the following reasons: (a) it is triggered by the recognition that an individual is in a state of emotional distress, and (b) it is aimed to alleviate this negative state. To alleviate the emotional distress, defending action involves a specific negative action against the aggressor to protect an individual victimized. Unlike the comforting action, defending behavior implies a direct action against the aggressor as a consequence of a negative evaluation of the aggressor's antisocial action. Furthermore, there is no doubt that defensive behavior, like other prosocial behaviors, contributes to social well-being, strengthening the positive peer group experiences across development (Dirks et al., 2018).

What is the underlying motivation for defensive behaviors? What elicits this behavior? How are these behaviors triggered in the first years of life? The present article aims to answer these questions by reviewing recent evidence on defending behaviors in the second year of life.

## Defending Behaviors

We think that defending requires the ability (i) to identify both another's an emotional state and the cause of the negative state (Saarni et al., 2007), (ii) to evaluate the aggressor's behavior as antisocial (Van de Vondervoort and Hamlin, 2016), and (iii) to recognize the negative behavior (defending action) toward to antisocial agent as positive (Hamlin et al., 2011).

Firstly, from the first days of life newborns demonstrate to respond to other's distress with the distress of their own (Sagi and Hoffman, 1976; Davidov et al., 2021), and within the first year of life, infants differentiate positive and negative emotions (Field et al., 1982, 1983; Walker-Andrews, 1997; Montague and Walker-Andrews, 2001; Farroni et al., 2007; Flom and Bahrick, 2007; Grossmann, 2010; Flom et al., 2018; for a review see also Ruba and Repacholi, 2020). Furthermore, several studies demonstrated that infants are significantly attuned to emotionally salient events, such as seeing someone in pain after hurting themselves (Zahn-Waxler et al., 1992). During these events, infants at 8 months will show increasingly observable nonverbal empathic responses, such as concern (e.g., Zahn-Waxler et al., 1992; Roth-Hanania et al., 2011), and later, at around 14 months, they will show more prosocial behaviors, such as comforting and helping (e.g., Warneken and Tomasello, 2007; Svetlova et al., 2010; Dunfield et al., 2011). Interestingly, Vaish et al. (2009) found that at 18 months, toddlers are more likely to engage in prosocial behaviors toward a victim, also in the absence of any distressing cues exhibited by the victim herself. Thus, at this age prosocial behaviors are elicited not only by the valence of the victim's emotional expressions but also simply by the valence of the events.

Secondly, based on some previous findings of infants' expectations, it is clear that preverbal infants possess specific socio-cognitive abilities to assess defensive behaviors. Infants showed to recognize the goals of an action performed by geometrical figures (Csibra, 2008) or human hands (Woodward, 1998), to discriminate between positive and negative interactions involving two agents (Premack and Premack, 1997), and to evaluate a puppet's helping or hindering action toward another one (Hamlin et al., 2007, 2011; Hamlin, 2013). Kanakogi et al. (2013) found that 10-month-old infants may discriminate the victim from the aggressor, showing a personal preference for the victim rather than for the aggressor. From the second year of life, toddlers can infer dominance from both agents' body sizes (Thomsen et al., 2011) and type of social interactions (Mascaro and Csibra, 2012). Taken together, these findings demonstrated an early capacity to distinguish social roles in laboratory conditions using artificial stimuli, often computer-presented animations showing a negative interaction between two agents. When infants were presented with an animation showing a triadic relation, they expected the third agent to intervene (placing himself in the middle between the victim and aggressor; Kanakogi et al., 2017) or to defend with a harmful action against the aggressor (e.g., pushing away; Geraci, 2020a). We distinguish defending behaviors from intervening behaviors only for different behavioral responses that are performed to stop aggressive interaction between two agents: the *intervener* stops the aggressive interaction with a *less negative* action against the aggressor, and the *defender* attacks the aggressor with a *more negative* action against the aggressor. The purpose of both actions is to protect the victim from the aggressor, but the behavior adopted is different. Furthermore, reflecting on the different behavioral responses of the defender and the intervener, it would seem that the defender desires to punish the aggressor more strongly (Hamlin et al., 2011; Meristo and Surian, 2014; Marshall et al., 2021).

On intervening behaviors, Kanakogi et al. (2017) investigated the developmental trajectory of perceiving and understanding protective third-party interventions in 6- and 10-month-old infants. Infants were presented with an event involving three agents: an aggressor strongly hitting a victim while a bystander watched from within an enclosure. This event concluded when the bystander interfered in that aggressive interaction by inserting itself or not inserting itself. In a subsequent reaching choice task, 6-month-old infants showed a personal preference for the interfering agent rather than the non-interfering agent. Additional experiments revealed the psychological processes underlying infants' choices: 6-month-olds understood the interfering agent want to protect the victim from the aggressor, but only 10-month-olds approved this intervention after considering the intentions of the interfering agent that were coherent with their prosocial expectations. These results revealed that 10-month-olds understand the intention behind intervening to prevent a victim from being harmed. On third-party defensive behaviors, a recent study found that 20-month-olds tend to reward and prefer those who defend a victim from an aggressor over who does not defend, and expect a bystander to approach the defender puppet rather than the non-defender (Geraci, 2020a). In Experiment 1, toddlers after seeing a puppy puppet played the role of victim, showed a preference for the defensive puppet over the non-defensive puppet, and also, they attributed the same preference to a bystander. Experiment 2 provides converging evidence in support of the first experiment, demonstrating that toddlers' expectations and evaluations of defensive behaviors are applied to victims independent of their size or age. Experiment 3 revealed that toddlers' evaluations guide awarding behaviors. These results revealed that toddlers understand and also evaluate defensive behaviors toward third parties, in line with previous findings on early social evaluations of helping behaviors (Hamlin et al., 2007; Hamlin, 2013; Van de Vondervoort and Hamlin, 2016; Surian and Franchin, 2017a), distributive behaviors (Geraci and Surian, 2011; Sloane et al., 2012; Surian and Franchin, 2017b; Ziv and Sommerville, 2017) and intervening behaviors (Kanakogi et al., 2017; Geraci, 2020b).

Other research investigates whether young children engage in defending actions. Previous studies found that preschool children defend themselves by protesting, arguing, and tattling (Dunn and Munn, 1987; Ingram and Bering, 2010) when they perceived themselves to be the victims of moral violations, such as property rights or physical harm. Also, 3-year-old children intervene in third-party moral transgressions, tattling on the transgressor, and act prosocially toward the victim (Vaish et al., 2011). In a review, Ingram (2014) claimed that during development, children tend to reduce physical aggression (direct aggression) that puts them at risk for retaliation and start to prefer tattling (indirect aggression). Very little is known about the emergence of direct aggression aimed to defend individuals victimized. Future studies are needed to investigate when infants or toddlers start to produce actively defending actions in their family toward siblings or their first peer groups.

## Conclusion

At this point, it is important to differentiate defending actions from helping, sharing, and comforting actions (Dunfield, 2014), considering their different eliciting factors (i.e., goal attribution > helping; material desire > sharing; emotional distress > comforting; emotional distress + punitive motivation > defending). The recent findings can be consistent with Hoffman's theoretical account (1982, 2000), according to which social, emotional, and cognitive development is an integral part of prosocial development. The ability to represent other's emotional distress alone is not sufficient for functional behavioral responses aimed to alleviate others' negative emotional states. Furthermore, the understanding of negative emotional states and their causes could involve an *intuitive, automatic, and emotion-based evaluation* (Van de Vondervoort and Hamlin, 2016), before generating a motivation to defend a victim from an attacker by engaging in aggressive behaviors. In this perspective, defensive behaviors could be considered a subtype of prosocial behaviors that can be elicited by recognizing another's emotional distress in Dunfield's smodel (2014), but in this case, it is necessary to evaluate the attacker as dangerous and evil to expect a bystander to engage a defensive behavior with an active action against the aggressor (Geraci, 2020a). Finally, defense behavior strongly inspires an evolutionary explanation of the emergence of a moral sense in terms of cooperation between our ancestors for their survival (Hamlin, 2013). Future studies should investigate toddlers' active behavioral engagements in defending actions to better explain better the emergence of prosocial behaviors, the link between the toddlers' aggressive behaviors (Vaish et al., 2009), and expectations of defending actions in third-party contexts (Geraci, 2020a).

## Author Contributions

All authors listed have made a substantial, direct and intellectual contribution to the work, and approved it for publication.

## Conflict of Interest

The authors declare that the research was conducted in the absence of any commercial or financial relationships that could be construed as a potential conflict of interest.
